# Practices and determinants of delivery by skilled birth attendants in Bangladesh

**DOI:** 10.1186/1742-4755-11-86

**Published:** 2014-12-11

**Authors:** Nazrul Islam, Mohammad Tajul Islam, Yukie Yoshimura

**Affiliations:** School of Population and Public Health, Faculty of Medicine, University of British Columbia, Room# 417, 2206 East Mall, Vancouver, BC V6T 1Z3 Canada,; Japan International Cooperation Agency, Dhaka, Bangladesh

**Keywords:** Skilled birth attendants (SBAs), Bangladesh, Determinant(s), Practice(s)

## Abstract

**Introduction:**

Utilization of Skilled Birth Attendants (SBAs) at birth is low (20%) in Bangladesh. Birth attendance by SBAs is considered as the “single most important factor in preventing maternal deaths”. This paper examined the practices and determinants of delivery by SBAs in rural Bangladesh.

**Methods:**

The data come from the post-intervention survey of a cluster-randomized community controlled trial conducted to evaluate the impact of limited post-natal care (PNC) services on healthcare seeking behavior of women with a recent live birth in rural Bangladesh (n = 702). Multivariable logistic regression model was used to identify the potential determinants of delivery by SBAs.

**Results:**

The respondents were aged between 16 and 45, with the mean age of 24.41 (± 5.03) years. Approximately one-third (30.06%) of the women had their last delivery by SBAs. Maternal occupation, parity, complications during pregnancy and antenatal checkup (ANC) by SBAs were the significant determinants of delivery by SBAs. Women who took antenatal care by SBAs were 2.62 times as likely (95% CI: 1.66, 4.14; p < 0.001) to have their delivery conducted by SBAs compared to those who did not, after adjusting for other covariates.

**Conclusion:**

Our findings suggest that ANC by SBAs and complications during pregnancies are significant determinants of delivery by SBAs. Measure should be in place to promote antenatal checkup by SBAs to increase utilization of SBAs at birth in line with achieving the Millennium Development Goal-5. Future research should focus in exploring the unmet need for, and potential barriers in, the utilization of delivery by SBAs.

**Electronic supplementary material:**

The online version of this article (doi:10.1186/1742-4755-11-86) contains supplementary material, which is available to authorized users.

## Background

Maternal deaths across the globe have been estimated to be approximately 289,000 in 2013, while the death toll of newborn within the first 4 weeks of birth has reached at 3.6 million [1–3].

Most of the complications related to pregnancy and childbirth are unpredictable, and take place around the time of delivery and postpartum period. This is why, access to Skilled Birth Attendants (SBAs) is strongly recommended for all the pregnant women so as to make sure a normal delivery is conducted well, related complications are recognized early and referred immediately to the appropriate healthcare facilities [4]. Birth attendance by SBAs is considered as the “single most important factor in preventing maternal deaths” [5]. Delivery attendance by SBAs is also very important in preventing stillbirths and improving newborn survival [4, 6]. The proportion of births attended by SBAs is one of the two indicators of the progress toward achieving Millennium Development Goal (MDG)- 5 [7].

The utilization of SBAs at birth is quite low in Bangladesh. About one in every five deliveries is attended by SBAs; the proportion is even lower in slum and tribal areas [8]. Home delivery assisted mainly by Traditional Birth Attendants (TBAs) is as high as 71% in Bangladesh [9, 10]. Government of Bangladesh initiated the Community-based Skilled Birth Attendant (CSBA) program to increase accessibility to skilled delivery at home in 2003 with the target to train 13,500 government field staff as CSBAs. As of June 2014, nearly 9,000 government CSBAs have been trained. Besides, some of the development partners have also been supporting the CSBA training to the private candidates, especially to cover the hard-to-reach areas. Recent evaluation of the CSBA program indicates that even though CSBAs are available in the rural areas, their utilization is low in the community [11]. It is, therefore, imperative to examine the determinants of delivery by SBAs so as to understand well the areas that require further policy reform and programmatic interventions in line with progress toward MDG-5. Determinants of delivery by SBAs identified in previous studies have been inconsistent, and there is paucity of information in Bangladeshi context. Current paper aims at identifying the determinants of delivery by SBAs in rural Bangladesh.

## Methods

The data analyzed in this study originated from the post-intervention survey of a cluster-randomized community trial (postnatal care intervention trial) conducted in Monohardi, a rural upazila (sub-district) of Narsingdi district, Bangladesh. The objective of the trial was to test the effectiveness of engaging community volunteers, working under the community support groups, in increasing the coverage of postnatal care by skilled healthcare providers. The study was implemented by the Government under the Safe Motherhood Promotion Project (SMPP) [12], supported by Japan International Cooperation Agency (JICA) in collaboration with a non-government organization, CARE-Bangladesh. Out of 11 unions (sub sub-district) of the upazila, 6 were randomly selected (3 for intervention and 3 for comparison) for the study. The intervention was implemented in the villages where community support groups were present.

Total population of the intervention and comparison areas was 42,370. The expected annual births were estimated as 957 based on crude birth rate of 22.6 per 1,000 population [9]. However, 702 women who had a live birth between November 2010 and October 2011 (study population) were identified through household visits and were interviewed by the data collectors.

Twelve female interviewers were recruited for the data collection. The interviewers were graduates from different disciplines with prior experience in data collection. Supervision and monitoring of data collection were done by the 4 SMPP project staff. All the data collectors and supervisors were trained for three days on the contents of the questionnaire, and data collection techniques. However, they were blinded about the objectives of the study. The Principal Investigator of the PNC study coordinated and supervised the whole efforts. Data were collected between 20 December 2011 and 1 January 2012.

A structured pre-coded questionnaire was used for face-to-face interview. This paper analyzed the data to explore the delivery care practices and factors associated with the delivery by SBAs in rural Bangladesh.

Descriptive statistics were presented to give an overview of the study participants. Bivariate analysis was done to find association between the explanatory variables and the outcome variable. Delivery by SBAs was defined as delivery conducted by qualified doctors, nurses, Community-based Skilled Birth Attendants (CSBAs), and Family Welfare Visitors (FWVs).

Odds ratio (OR) and 95% Confidence Intervals (CI) were calculated as effect measure. The variables found to be associated with the dependent variable at 10% level of significance in bivariate analysis was included in the multivariable Logistic Regression model to adjust for confounding effects after checking for multicollinearity. All the tests were two-sided, and a p-value of less than 0.05 was considered as significant. Data were analyzed in SPSS (version 20.0), and STATA (SE 12.1) [13].

### Ethical consideration

This study used secondary data generated in an intervention study. Both the intervention and analysis of data for this study were approved by the Directorate General of Health Services (DGHS). DGHS is the implementing and coordinating body of all the health related activities in Bangladesh under the Ministry of Health and Family Welfare. Verbal informed consent was obtained from the respondents before data collection.

## Results

**Figure 1:**
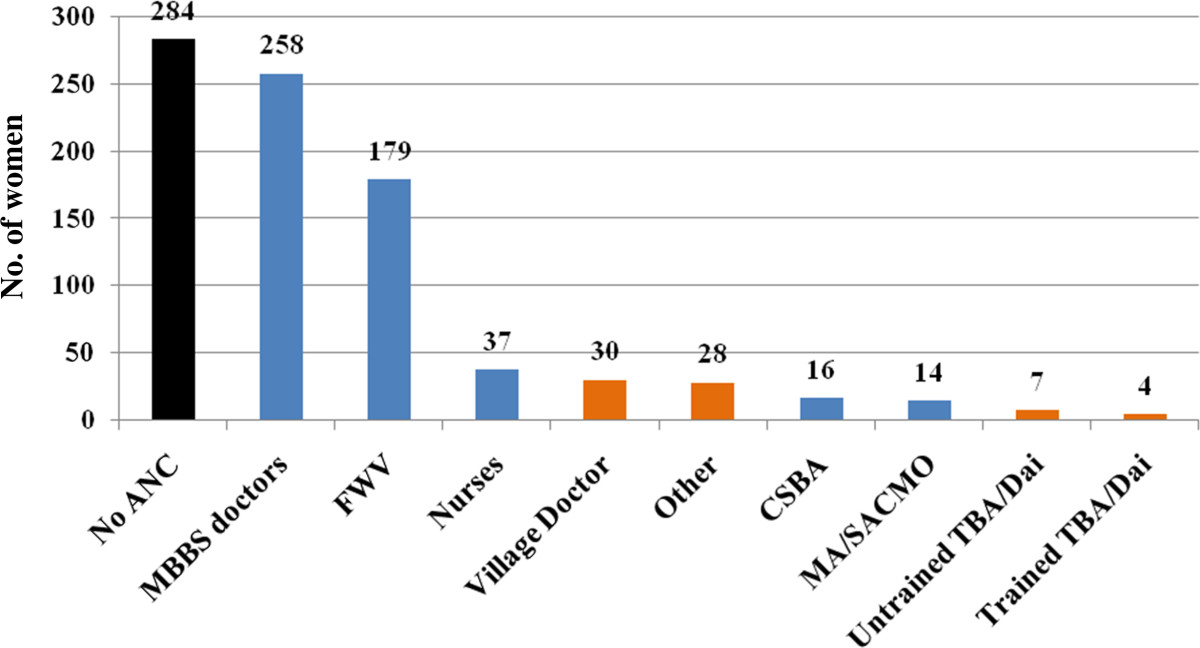
**Antenatal care provider to the respondents (multiple responses were there).**

**Figure 2:**
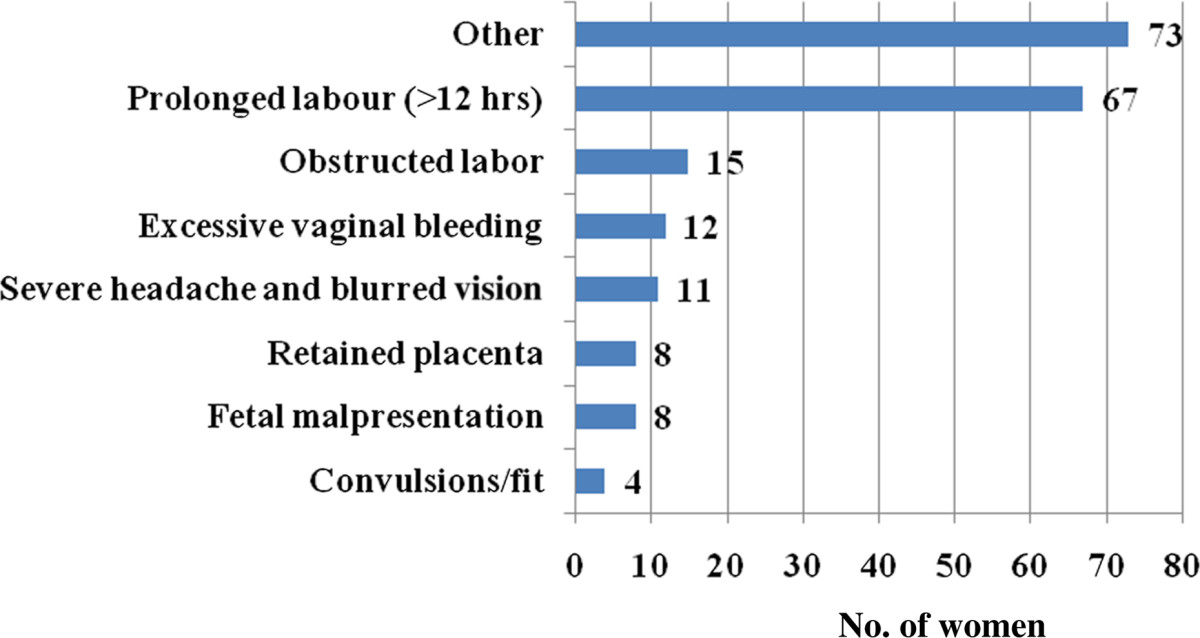
**Complications experienced during delivery by the participants (multiple responses were there).**

**Figure 3:**
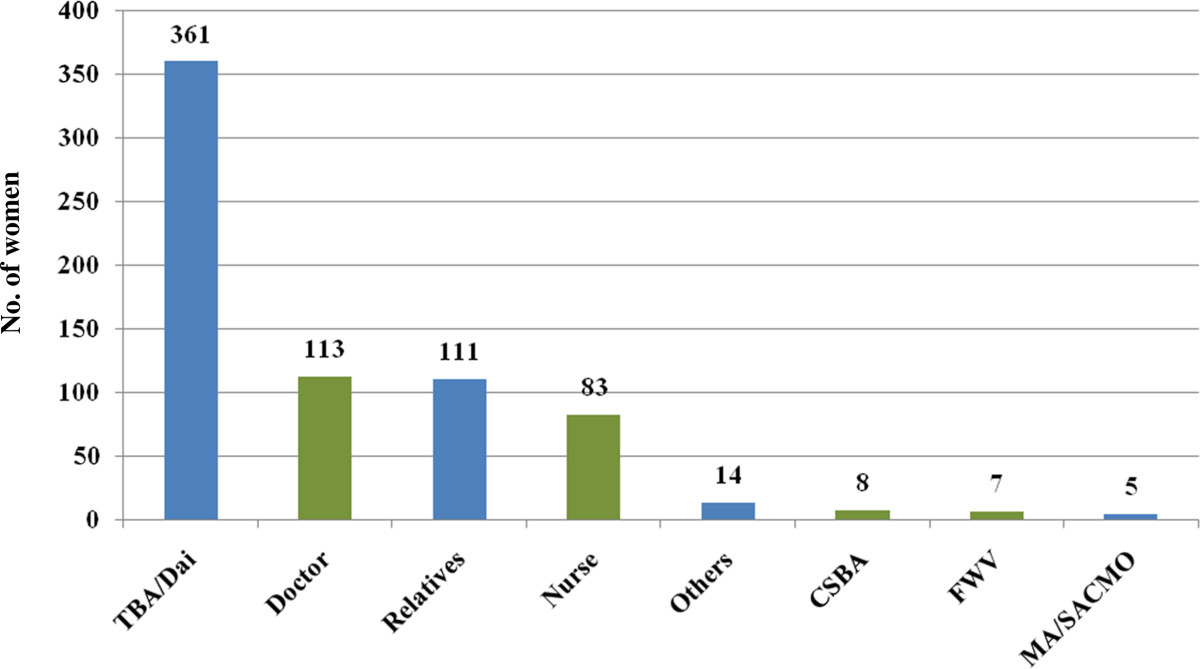
**Delivery providers.**

In total, 702 women were interviewed who had a live birth between November 2010 and October 2011. Approximately one-third (30.06%) of the respondents had their last delivery by SBAs. Table 1 summarizes the socio-demographic characteristics of the respondents. The mean age of the women was 24.41 (± 5.03) years, mostly between the ages of 20 and 34 years. Approximately 14% experienced teenage pregnancy. Almost 90% of the respondents were Muslim while 95% were housewives. More than 60% of the participants were educated up to secondary level and beyond, while about 15% never attended school. About half the participants’ husbands completed primary schooling and continued to secondary education; however, a quarter of them did not attend school ever. Monthly family income ranged between 1,500 and 80,000 BDT (Bangladeshi Taka; 1 USD ≈ 78.55 BDT) with median income of 8,000 BDT (≈US$ 100). About 40% of the respondents had one or no child before this pregnancy. About 60% of all the respondents took antenatal care (ANC) by SBAs, and almost a quarter (n = 169) of the respondents experienced complications during pregnancy. About three-fourth (73.65%; n = 517) of the women had their delivery at home while 84.76% (n = 595) had normal vaginal delivery.Figure 1 shows the antenatal care practice of the respondents. A substantial proportion (>40%) of women did not seek antenatal care from SBAs, or did not seek for any antenatal care at all. Figure 2 demonstrates types of complications the respondents experienced during the last pregnancy. Prolonged labor (labor pain for more than 12 hours) was the single leading complication. Figure 3 shows that a substantial proportion of delivery was conducted by traditional birth attendants (TBAs).

**Table 1:** **Socio-demographic characteristics of the respondents**

Characteristics	No.	%
**Age in years**		
<20	98	13.96
20-34	563	80.20
≥35	41	5.84
Mean (SD)	24.41 (5.03)	
**Religion**		
Islam	625	89.03
Hinduism	77	10.97
**Women’s education**		
No education	105	14.96
1-5 years	164	23.36
≥6 years	433	61.68
**Women’s occupation**		
Housewife	667	95.01
Others	35	4.99
**Husband’s education**		
No education	180	25.90
1-5 years	177	25.47
≥6 years	338	48.63
**Husband’s occupation**		
Laborer	430	61.25
Jobs including professionals	68	9.69
Business/Living abroad	204	29.06
**Monthly family income (BDT)**		
≤ 6,000	196	27.92
6,001-10,000	313	44.59
>10,000	193	27.49
**Parity (Number of children)**		
0 or 1	276	39.32
2 or more	426	60.68
**Any ANC by SBAs**	409	59.02
**Complications faced during pregnancy**	169	24.07

BDT = Bangladeshi Taka; ANC = Antenatal Care; SBAs = Skilled Birth Attendants.

Associations between respondents’ socio-demographic characteristics and pregnancy-related behaviors (e.g., antenatal care by skilled healthcare providers, pregnancy-related complications etc.) and delivery by skilled birth attendants are summarized in Table 2. It shows that women’s occupation, husband’s occupation, parity, antenatal care (ANC) by SBAs, and complication during pregnancy were significantly associated with delivery by SBAs in bivariate analysis.

**Table 2:** **Association between socio-demographic characteristics and pregnancy-related behavior and delivery by skilled birth attendants**

	Delivery by SBAs			
Characteristics	Yes (n = 211)	No (n = 491)	OR (95% CI)	p-value
**Age in years**				
<20	33 (15.64)	65 (13.24)	1.22 (0.78, 1.93)	0.384
20-34	165 (78.20)	398 (81.06)	Ref.	
≥35	13 (6.16)	28 (5.70)	1.12 (0.57, 2.22)	0.745
**Religion**				
Islam	188 (89.10)	437 (89.00)	Ref.	
Hinduism	23 (10.90)	54 (11.00)	0.99 (0.59, 1.66)	0.970
**Women’s education**				
No education	23 (10.90)	82 (16.70)	Ref.	
1-5 years	29 (13.74)	135 (27.49)	0.77 (0.42, 1.41)	0.393
≥6 years	159 (75.36)	274 (55.80)	2.07 (1.25, 3.42)	0.005
**Women’s occupation**				
Housewife	190 (90.05)	477 (97.15)	Ref.	
Others	21 (9.95)	14 (2.85)	3.77 (1.88, 7.56)	<0.001
**Husband’s education**				
No education	34 (16.11)	146 (30.17)	Ref.	
1-5 years	43 (20.38)	134 (27.69)	1.38 (0.83, 2.29)	0.215
≥6 years	134 (63.51)	204 (42.15)	2.82 (1.83, 4.35)	<0.001
**Husband’s occupation**				
Laborer	93 (44.08)	337 (68.64)	Ref.	
Professionals	36 (17.06)	32 (6.52)	4.07 (2.40, 6.92)	<0.001
Business/Living abroad	82 (38.86)	122 (24.85)	2.44 (1.70, 3.50)	<0.001
**Monthly family income (BDT)**				
≤ 6,000	42 (19.91)	154 (31.36)	Ref.	
6,001-10,000	83 (39.34)	230 (46.84)	1.32 (0.87, 2.02)	0.195
>10,000	86 (40.76)	107 (21.79)	2.95 (1.89, 4.59)	<0.001
**Parity (Number of children)**				
0 or 1	102 (48.34)	174 (35.44)	Ref.	
2 or more	109 (51.66)	317 (64.56)	0.59 (0.42, 0.81)	0.001
**Any ANC by SBAs**				
Yes	164 (78.47)	245 (50.62)	3.56 (2.44, 5.17)	<0.001
No	45 (21.53)	239 (49.38)	Ref.	
**Complication during pregnancy**				
Yes	121 (57.35)	48 (9.78)	12.41 (8.29, 18.58)	<0.001
No	90 (42.65)	443 (90.22)	Ref.	

The results of multivariable logistic regression are summarized in Table 3. It shows that, after adjustment for covariates in the model, the variables found to be significantly associated with delivery by SBAs were non-housewives (OR: 3.08; 95% CI: 1.27, 7.51; p = 0.013), having 2 or more children (OR: 0.61; 95% CI: 0.40, 0.93; p = 0.023); ANC by SBAs (OR: 2.62; 95% CI: 1.66, 4.14; p < 0.001), and complications during pregnancy (OR: 15.00; 95% CI: 9.39, 23.95; p < 0.001).

**Table 3:** **Determinants of delivery by skilled birth attendants from multivariable logistic regression**

	Delivery by SBAs	
Characteristics	Adjusted OR (95% CI)	p-value
**Women’s education**		
No education	Ref.	
1-5 years	0.54 (0.25, 1.20)	0.131
≥6 years	0.98 (0.47, 2.03)	0.950
**Women’s occupation**		
Housewife	Ref.	
Others	3.08 (1.27, 7.51)	0.013
**Husband’s education**		
No education	Ref.	
1-5 years	0.98 (0.51, 1.90)	0.958
≥6 years	1.36 (0.73, 2.54)	0.335
**Husband’s occupation**		
Laborer	Ref.	
Professionals	1.87 (0.92, 3.82)	0.086
Business/Living abroad	1.63 (1.02, 2.61)	0.043
**Monthly family income (BDT)**		
≤ 6,000	Ref.	
6,001-10,000	1.36 (0.79, 2.35)	0.269
>10,000	2.32 (1.26, 4.29)	0.007
**Parity (Number of children)**		
0 or 1	Ref.	
2 or more	0.61 (0.40, 0.93)	0.023
**Any ANC by SBAs**		
Yes	2.62 (1.66, 4.14)	<0.001
No	Ref.	
**Complication during pregnancy**		
Yes	15.00 (9.39, 23.95)	<0.001
No	Ref.	

## Discussion

In this study, proportion of women who took any antenatal care by SBAs was 59.02%, which is higher than the earlier estimates of 48.7% in 2007 and 46.4% in 2011 [9, 14]. Approximately one-third (30.06%) of the participants had their last delivery by SBAs which is higher than the 2006 estimates of 14%, 2007 estimates of 13.2% [14], 2009 estimates of 19.2% [15], and 2011 estimates of 25.2%, but little less than another estimates of 35% in 2006 [16]. Several factors have been identified as potential determinants of delivery by SBAs. In our study, maternal occupation, parity, complications during pregnancy and antenatal checkup by SBAs were statistically significant determinants of delivery by SBAs. Monthly family income was significant only when it was more than 10,000 BDT/month. Some of our findings are compatible with the findings from other studies while others are not. For example, complications during pregnancy, ANC visits (2 or more), and asset quintile were found to be significant determinants for delivery by SBAs (latter showing a dose–response relationship) in Bangladesh, while women’s education was not found to be a significant determinant [16]. All these findings are compatible with our study. However, this study [16] also found that religion is a significant determinant of delivery by SBAs which is not compatible with our findings.

Maternal age was found either to have no effect or to increase the utilization of SBAs at birth in several studies. Marital status was found to have no association with delivery by SBAs [17–19]. Studies also identified racial [20], and ethnic and/or religious [21–26] disparities in the utilization of SBAs, unlike the findings of our study. Many studies found women’s and their husbands’ education as potential determinants of delivery by SBAs [4, 27], which was not evident in our study, nor in another study by Anwar et al. [16]. Other significant determinants of delivery by SBAs include ANC [4, 27–29], parity/birth order [30, 31], complications during pregnancy [4, 27], and maternal occupation [32] among others.

ANC has also been found to be associated with increased institutional delivery, and improved perinatal survival [33]. Physical accessibility to skilled birth attendants has also been identified as potential determinant of delivery by SBAs [4]. However, data on these are missing in our study which is one of the major limitations of our study. Other possible reasons for poor utilization of SBAs during delivery may include: insufficient community awareness regarding the usefulness of utilization of SBAs during the delivery, lack of appropriate information with regard to availability and delivery services provided by SBAs etc. [27, 29]. However, health information awareness program targeting women (and their husbands) may not be sufficient in improving the utilization of SBAs since women, and in most cases even their husbands, are not included in decision-making process even when the issues primarily pertain to them [27]. Mothers and mother-in-laws should also be included in the awareness campaign since they are often the decision makers in the issues related to delivery [27].

Of all the deliveries by SBA, majority (85.78%) were conducted at the health facilities. Only 5.8% of the home deliveries were conducted by SBAs. Government of Bangladesh introduced CSBA training program in 2003 in collaboration with World Health Organization (WHO) and United Nations Population Fund (UNFPA) [11]. The goal of the program is to provide at least 2 CSBAs in each of the unions. About 7,000 CSBAs have been trained until 2012. The CSBA evaluation conducted in 2011 shows that the utilization of CSBAs are poor for home deliveries. Several problems have been identified for this, which include busy schedule of the service providers trained as CSBA, inadequate monitoring and supervision, and lack of community awareness. Other studies also indicate lack of awareness of community about CSBA in the locality. These findings indicate that promotional activities are required to improve utilization of CSBAs by the community for home delivery.

Finally, according to Millennium Development Goal (MDG) 5 target set for Bangladesh by 2015, delivery by skilled birth attendants has to be increased to 50%, and (at least one) antenatal care coverage has to be increased to 71.2%. However, if the government has to achieve the MDG target of at least 50% deliveries by SBAs, either institutional delivery has to be increased or home delivery by SBA has to be increased. About 3 million births take place annually in Bangladesh. A 20% increase of institutional delivery means that at least an additional 600,000 deliveries need to be conducted at the health facilities. Though rural health facilities, in general, are under-utilized, the health facilities are not prepared to accommodate such a huge number of deliveries at this moment. On the other hand, to promote home delivery by SBA, mass awareness of community and physical accessibility to CSBAs need to be considered. The findings from this study, supported by those from other studies, suggest that Bangladesh has to exert much concerted effort to meet the indicators of MDG-5 [34–36].

## Conclusion

The proportion of deliveries by SBAs was similar to the estimates of nationally representative survey findings though the proportion of women received any ANC was higher. Maternal occupation, parity, complications during pregnancy and antenatal checkup by SBAs were the significant determinants of delivery by SBAs. Promotion of quality antenatal care in the community may improve the delivery by skilled providers in rural areas of Bangladesh. Further research to explore the unmet need for, and potential barriers in, the utilization of skilled birth attendants for delivery is recommended.

## Authors’ original submitted files for images

Below are the links to the authors’ original submitted files for images. Authors’ original file for figure 1 Authors’ original file for figure 2 Authors’ original file for figure 3

## Abbreviations

ANC: Antenatal Care
CI: Confidence Intervals
SACMO: Sub-assistant Community Medical Officer
CSBA: Community Skilled Birth Attendant
FWV: Family Welfare Visitor
JICA: Japan International Cooperation Agency
MBBS: Bachelor of Medicine and Surgery
MDG: Millennium Development Goal
PNC: Postnatal Care
SBA: Skilled Birth Attendant
TBA: Traditional Birth Attendant
UNFPA: United Nations Population Fund
WHO: World Health Organization.
