# Supplementary figures and images for: Practices and determinants of delivery by skilled birth attendants in Bangladesh
Source: Reprod Health. 2014 Dec 11;11:86. doi: 10.1186/1742-4755-11-86 (PMC4292816; doi:10.1186/1742-4755-11-86)

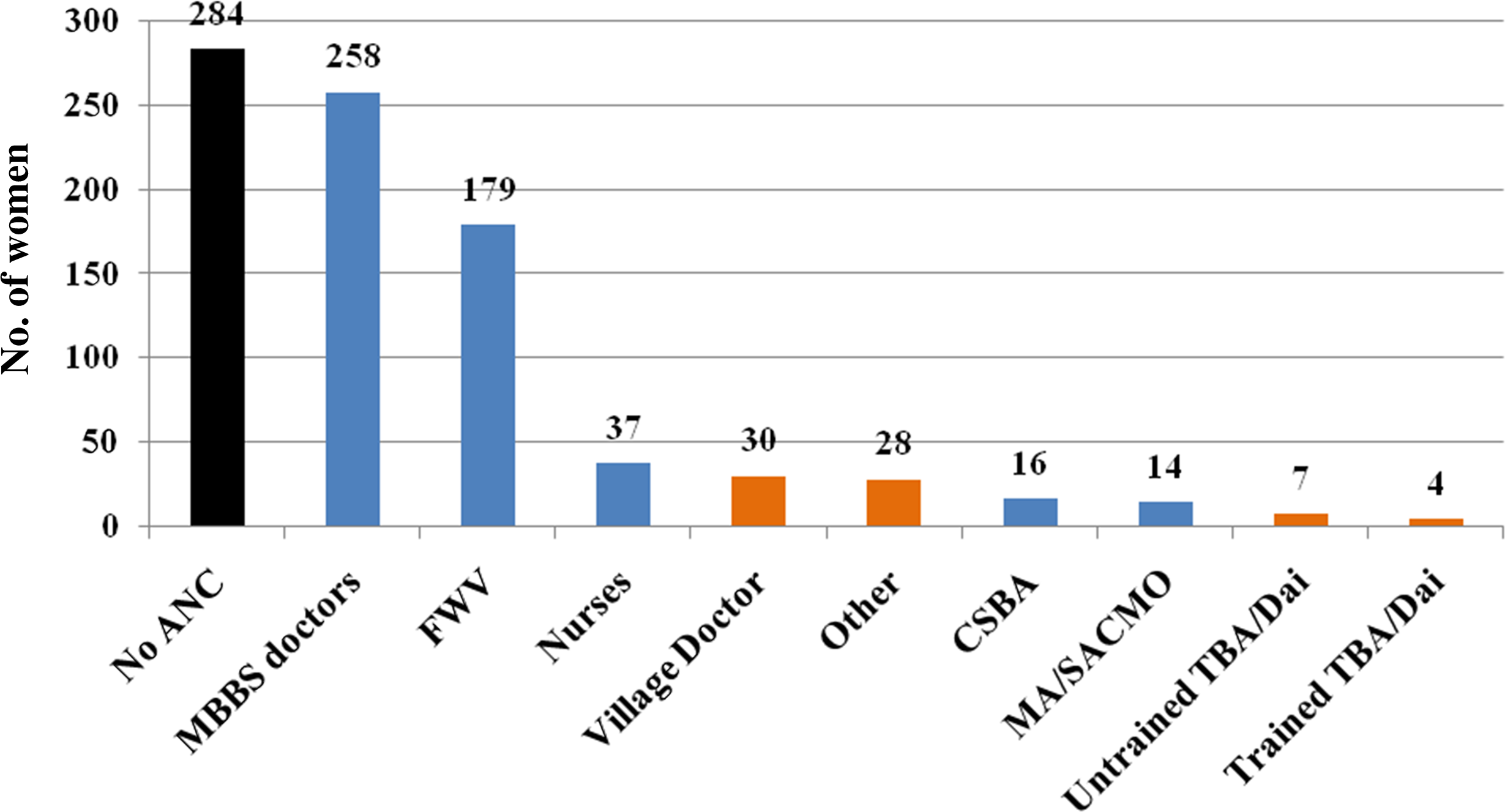

Supplement: Supplementary file 1 — Authors’ original file for figure 1 [file 12978_2014_338_MOESM1_ESM.tiff]

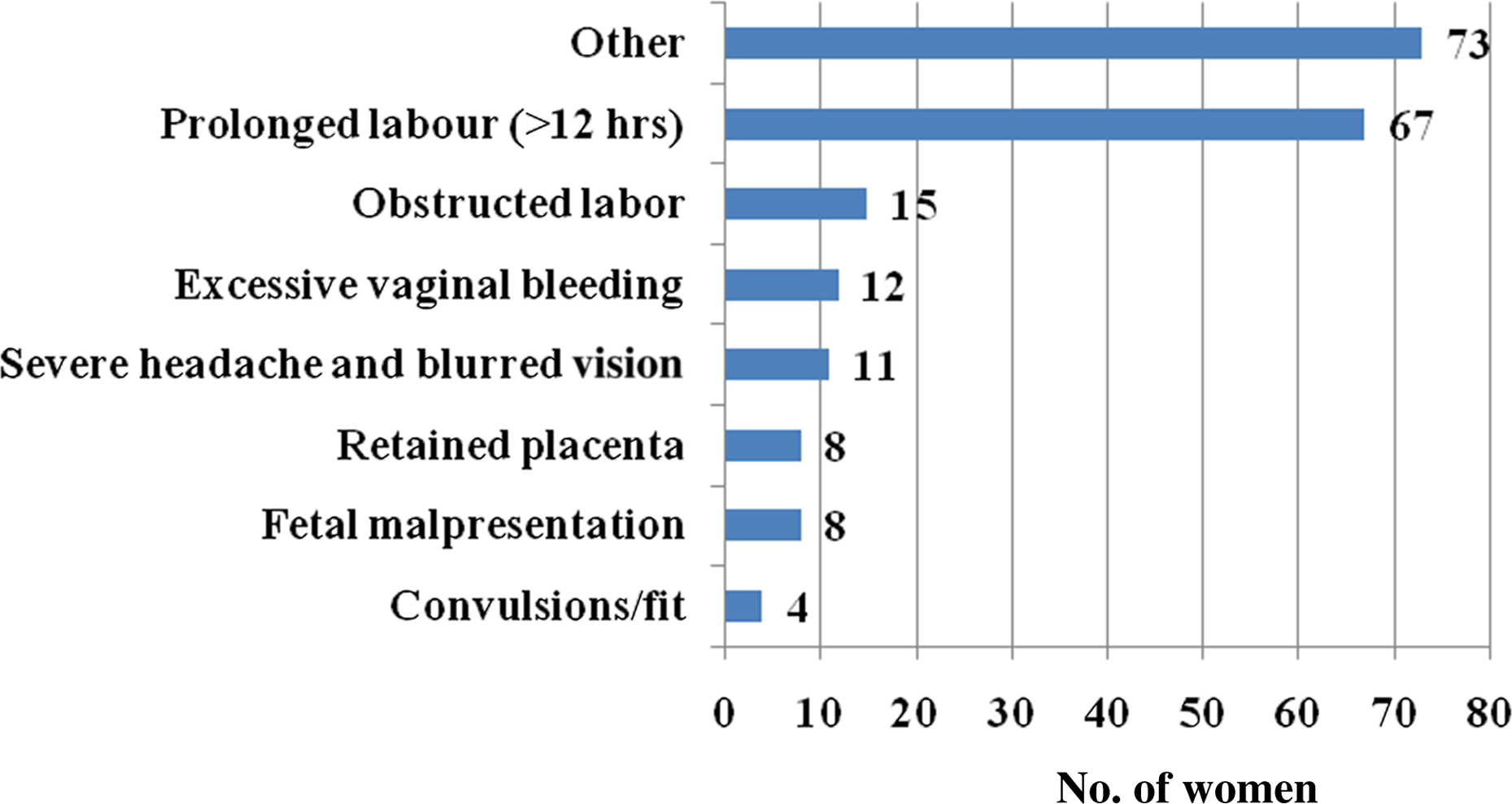

Supplement: Supplementary file 2 — Authors’ original file for figure 2 [file 12978_2014_338_MOESM2_ESM.tiff]

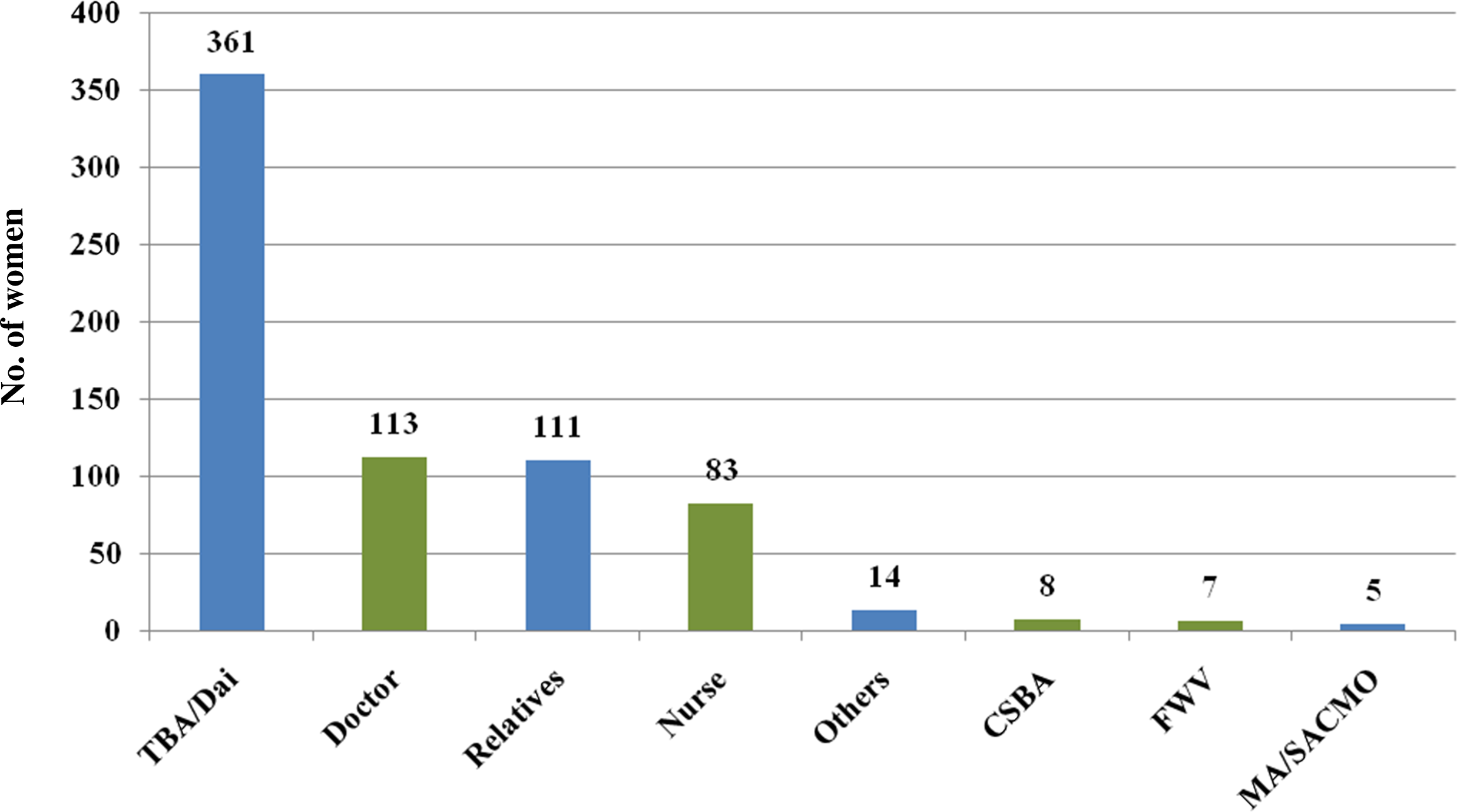

Supplement: Supplementary file 3 — Authors’ original file for figure 3 [file 12978_2014_338_MOESM3_ESM.tiff]
